# Polyphenol-Rich *Larix decidua* Bark Extract with Antimicrobial Activity against Respiratory-Tract Pathogens: A Novel Bioactive Ingredient with Potential Pharmaceutical and Nutraceutical Applications

**DOI:** 10.3390/antibiotics10070789

**Published:** 2021-06-28

**Authors:** Marta Faggian, Giulia Bernabè, Sara Ferrari, Stefano Francescato, Gianni Baratto, Ignazio Castagliuolo, Stefano Dall’Acqua, Gregorio Peron

**Affiliations:** 1 Unired srl, Via Niccolò Tommaseo 69, 35131 Padova, Italy; nutraceutica@unired.it; 2 Department of Molecular Medicine, University of Padova, Via Gabelli 63, 35121 Padova, Italy; giulia.bernabe@edu.unife.it (G.B.); ignazio.castagliuolo@unipd.it (I.C.); 3 Unifarco spa, Via Cal Longa 62, 32035 Santa Giustina, Belluno, Italy; sara.ferrari@unifarco.it (S.F.); stefano.francescato@unifarco.it (S.F.); gianni.baratto@unifarco.it (G.B.); 4 Department of Pharmaceutical and Pharmacological Sciences, University of Padova, Via Marzolo 5, 35131 Padova, Italy; stefano.dallacqua@unipd.it

**Keywords:** *Larix decidua* bark extract, procyanidins, polyphenols, green extraction, antimicrobial agent

## Abstract

Larch (*Larix decidua*) bark is a sawmill waste, traditionally used for antiseptic, expectorant and dermatological (wound healing, eczema, psoriasis) purposes. In this work, we developed a food-grade dry larch bark extract (LBE) from sawmill by-products using hydro-alcoholic extraction. The antibacterial activity of LBE was evaluated against respiratory-tract pathogens, i.e., *Staphylococcus aureus*, *Streptococcus pyogenes*, *Streptococcus pneumoniae*, *Klebsiella pneumoniae*, *Pseudomonas aeruginosa* and *Haemophilus influenza*, and it was compared to that of grapefruit seed extract (GSE), a commercially available raw material commonly proposed as antibacterial ingredient for over-the-counter products. Procyanidins (PACs) and other polyphenols contents in LBE were determined by HPLC-FLD-MS and HPLC-DAD-MS^n^, respectively. The antimicrobial activity of LBE and GSE was assessed using the micro-plate dilution technique in concentration range of 2–200 µg/mL, and the safety of these dosages was assessed in cellular and animal models. LBE showed considerable contents of PACs (15% *w*/*w*; especially B-type) and other polyphenols (3.8% *w*/*w*), among which the characteristic spiropolyphenols larixinol and epilarixinol were identified, together with the flavonoids isoquercitrin and rutin, already reported as growth inhibitors of different respiratory-tract pathogens. LBE showed higher antimicrobial activity compared to GSE, demonstrated by a growth inhibition range of 10–40% towards five of six strains tested, compared to 10–15% of GSE. These results suggest that LBE may represent a natural and sustainable source of active compounds with antibacterial activity for pharmaceutical and nutraceutical applications.

## 1. Introduction

European larch (*Larix decidua*) is a deciduous conifer typically found in mountainous areas of central Europe, and commonly used for carpentry, hydraulic and naval applications [1]. Larch bark represents a processing waste of wood industry and is commonly recycled for pellet fuels production and horticulture scopes. Larch bark is also used in traditional medicine as diuretic, expectorant, antiseptic and for the treatment of purulent wounds, chronic eczema and psoriasis [2].

In our previous work, we assessed the chemical composition and the antioxidant activity of different larch bark hydro-alcoholic dry extracts, processed with green extraction techniques [3]. Overall, results indicated that larch bark is a valuable source of antioxidant compounds like flavonoids and B-type procyanidins (PACs), and it may represent an innovative and sustainable ingredient for pharmaceutical, nutraceutical and cosmetic purposes. In the present work, a deeper investigation of the antimicrobial properties of a larch bark hydro-alcoholic dry extract (LBE) obtained by sawmill by-products is reported, specifically against respiratory-tract pathogens. Acute respiratory-tract infections are among the most diffused bacterial affections in humans, and one of the most common causes of antibiotic prescription [4]. Furthermore, lower and upper respiratory-tract infections are major causes of morbidity and mortality in children worldwide [5,6]. Like the gut, the upper respiratory-tract represents a reservoir of bacterial species that can play protective roles or be potentially pathogenic under certain conditions, and among these latter, *Streptococcus pyogenes*, *Haemophilus influenzae*, and *Staphylococcus aureus* have been reported as the most common, especially in children [7]. Overall, the treatment of bacterial infections is becoming increasingly challenging, considering that the unprecedented use of antibiotics is leading to the development of bacterial resistance, with dramatic consequences for human healthcare [8]. This leads to the urgent need to discover and develop novel antibiotics, and natural products could represent valuable sources to this regard. Nutraceuticals, defined as bioactive products isolated from herbal and alimentary sources and used for health purposes other than nutrition [9], could also represent valuable options to counteract respiratory-tract bacterial infections. In fact, these products can be used to enrich the diet with appropriate biologically active elements, allowing to obtain important health benefits beyond the normal nutritional effects, especially in the pediatric field [10].

Many plant-derived phenolic compounds have been reported to exert antimicrobial and anti-biofilm activities against a wide range of microorganisms, along with low toxicity and safety of use [11,12,13]. The antimicrobial properties of PACs and hydrolysable tannins of variable natural sources, for example, are well recognized. These compounds affect bacterial activity with different mechanisms of action, such as enzymatic inhibition, removal of essential substances, metal ions complexations, breakdown of membrane cells, direct interference with microbial metabolism and adhesion capacity [14,15,16,17]. Li et al. reported that the mechanisms of action of PACs from larch (*Larix gmelinii*) bark against *S. aureus* involve the alteration of bacterial morphology, membrane function, genetic expression and metabolic activities [18]. Among other phenolic constituents frequently characterized in plant extracts, flavonoids and stilbenes have been reported as effective antimicrobial agents [19,20]. Constituents of the methanol extract of *L. decidua* bark, specifically the flavonoid kaempferol and the stilbene astringin, have been associated to its antimicrobial activity against *S. aureus*, although possible mechanisms of action have not been elucidated [21].

The antimicrobial properties of various conifers have been already described in literature. *Pinus pinaster* bark extract has been reported to exert bacteriostatic activity against both Gram-positive and Gram-negative strains [22], while larch wood and pine sapwood have been reported to be effective against *S. aureus*, *Bacillus subtilis* and *Enterococcus faecium*, respectively [23]. Salem et al. evaluated the antimicrobial activity of *L. decidua* and *Picea abies* bark and wood methanol extracts against several fungi and bacterial strains, showing MIC (minimum inhibitory concentration) and MBC (minimum bactericidal concentration) for *L. decidua* only slightly higher than common antibiotics, i.e., 0.11–0.54 mg/mL and 0.36–0.96 mg/mL respectively [24]. Hubert et al. evaluated the antimicrobial activity of extracts obtained from ten conifer barks, including *L. decidua*, against *S. aureus*. Results indicated that methanol and methanol/water extracts were more efficient than n-heptane extracts, with *Quercus rubur*, *L. decidua* and *P. abies* being the most active species [25].

In this study, as a continuation of our previous work on *L. decidua* [3], a standardized LBE obtained by bark bio-waste was developed using an industrial pilot plant, with the aim to evaluate this product as natural antimicrobial ingredient for pharmaceutical and nutraceutical applications. For this purpose, its antibacterial activity against respiratory-tract pathogens such as *Staphylococcus aureus*, *Streptococcus pneumoniae*, *Klebsiella pneumoniae*, *Pseudomonas aeruginosa*, *Haemophilus influenzae* and *Streptococcus pyogenes* was investigated and compared with a commercial grapefruit (*Citrus paradisi*) seed extract (GSE), a raw material widely proposed in the last years as antimicrobial ingredient for cosmetics and nutraceuticals [26]. Furthermore, the PACs content of LBE was characterized by liquid chromatography coupled to fluorescence detector and mass spectrometry (HPLC-FLD-MS), and the phenolic profile was quantitatively and qualitatively evaluated by liquid chromatography coupled to diode array detector and multi-stage tandem mass spectrometry (HPLC-DAD-MS^n^).

## 2. Materials and Methods

### 2.1. Materials

Larch bark was provided by an Italian sawmill from Forno di Zoldo, Belluno, Italy. A commercial GSE D/E 4:1 was provided by an Italian distributor. GSE polyphenols were analyzed by HPLC-DAD using an internal method (reported in Supplementary Materials) and flavanone glycosides ascribable to *Citrus* species were reported to be 0.95% *w*/*w*, expressed as hesperidin.

Standard PAC-B2 was purchased from Extrasynthese (Genay, France). Methanol, HPLC-grade acetonitrile and HPLC-grade formic acid were purchased from Sigma Aldrich. Deionized water used in HPLC analyses was filtered through a Milli-Q system equipped with a 0.22-μm cut-off filter (Millipore, Burlington, MA, USA). Methicillin-resistant *S. aureus* (MRSA; ATCC 33592), *S. pyogenes* (ATCC 19615), *S. pneumoniae* (ATCC 33400), *K. pneumoniae* (ATCC BAA-1705), *P. aeruginosa* (ATCC 15692) and *H. influenzae* (ATCC 19418) were obtained from American Tissue Culture Collection (ATCC). Brain heart infusion (BHI), thioglycolate, and trypticase soy (TSB) broths were purchased from Difco Laboratories (Detroit, MI, USA). To culture *H. influenzae*, hemin (10 µg/mL) and βNicotinamide Adenine dinucleotide (βNAD; 2 µg/mL) were added to BHI. Instead, 10% *v*/*v* heat-inactivated fetal bovine serum (FBS) was added to BHI to culture *S. pyogenes*.

### 2.2. Extraction Procedure

Larch bark was dried at 40 °C for 40 h to a final humidity of 5%. The development of a sustainable extraction procedure for larch bark has been already reported in [3]. Briefly, the dried bark material was grinded to a fine powered (d = 300 µm) and extracted using 40% ethanol/water mixture under stirring for 60 min at room temperature, with a drug: solvent ratio of 1:10. The extract was then centrifuged using an industrial centrifuge and concentrated under vacuum using a vacuum evaporator. Finally, solvent was removed to dryness using a vacuum oven. The dry extract was then milled to 250 µm granulometry. The final drug:extract ratio was 7.5:1, and the extraction yield was 10%.

### 2.3. Cytotoxicity Assay

For cytotoxicity assay, adenocarcinomic human alveolar basal epithelial cells (A549 cell line) were cultured in Dulbecco’s Modified Eagle’s Medium (DMEM) supplemented with 10% FBS and 1% penicillin/streptomycin. Cells were seeded in 96-well microplates in complete medium, and LBE and GSE were added to a final concentration ranging from 0–2000 µg/mL. After 24 h at 37 °C, the culture medium was removed and replaced with fresh complete medium. Following additional 24 h incubation at 37 °C, 3-(4,5-dimethylthiazol-2-yl)-2,5-diphenyl tetrazolium bromide (MTT) solution (5 mg/mL) was added to each well, and cells incubated for 4 h at 37 °C. Formazan crystals were solubilized in 100 µL of sodium dodecyl sulphate 10% *w*/*v* and HCl 0.01 N, and the absorbance was recorded 16 h later at *λ* = 590 nm using a microplate reader (Sunrise Tecan, Männedorf, Switzerland). All experiments were repeated at least three times with triplicate determinations for each condition.

### 2.4. Acute Toxicity Study

Animal studies were performed by BSL BIOSERVICE Scientific Laboratories (Munich, Germany) and they were registered under the code STUIU20AA0028-1. All experimental protocols complied with the requirements of European Directive 2010/63/EU.

Six female Wistar rats (8–10 weeks old) were treated with LBE by oral gavage administration at a dosage of 2000 mg/kg body weight. LBE was suspended with the vehicle (corn oil; Sigma Aldrich, lot n. MKCK6411) at a concentration of 200 mg/mL and administered at a dose volume of 10 mL/kg. Prior to the administration, food was withheld from the test animals for 17 to 18 h (access to water was permitted).

All animals used in the study after their entrance at biosafety levels (BSL) were allowed to acclimatize to the laboratory conditions (temperature: 22 ± 3 °C; relative humidity: 55 ± 10%; artificial light sequence: 12 h light, 12 h dark) for at least 5 days. The animals were observed on delivery, on inclusion in the study and before administration for mortality/morbidity and other clinical signs. All animals were examined for clinical signs several times on the day of dosing and once daily until the end of the observation period (15 days). Their body weights were recorded on day 1 (prior to the administration) and on days 8 and 15 (data are reported in the Supplementary Table S1). At the end of treatment, all animals were necropsied and examined macroscopically.

### 2.5. Evaluation of Antimicrobial Activity against Respiratory-Tract Pathogens

Antimicrobial activity of LBE and GSE was determined by the microplate dilution method already reported in [27], with slight modifications. Bacteria were cultured in proper broths (MRSA, *K. pneumoniae*, and *P. aeruginosa* in TSB; *S. pyogenes* in BHI supplemented with 10% *v*/*v* FBS; *S. pneumoniae* in thioglycolate broth; *H. influenzae* in BHI supplemented with βNAD and hemin) at 37 °C. After 16 h they were collected by centrifugation and a bacterial suspension at optical density (OD) of 0.5 McFarland (1.5 × 10^8^ CFU/mL) was prepared. Then, 100 µL of properly diluted bacteria were inoculated in 96-well microplates. Stock solutions of LBE and GSE were prepared and added to microplates in a concentration range of 2–200 µg/mL. Microplates were incubated at 37 °C with horizontal shaking. Optical density at 620 nm was recorded at baseline and after 16 h incubation to assess the effect of extracts on bacterial growth. The percentage of survivors was presented with respect to the control mixture without any extract.

### 2.6. HPLC-FLD-MS Analysis of Larch Bark Procyanidins

LBE (50 mg) and dried larch bark (500 mg) were suspended in methanol (25 mL) and were extracted using an ultrasonic bath for 30 min. Samples were centrifuged at 13,000 revolutions per minute (rpm) for 10 min and liquids were collected in vial for analysis.

Analyses of PAC-A, PAC-B and polymerization degree were performed by HPLC-FLD-MS as previously reported [3,28]. Briefly, the chromatographic system was composed of an Agilent 1260 series quaternary pump (Agilent Technologies, Santa Clara, CA, USA) coupled to an Agilent 1260 fluorescence detector (FLD) and a Varian 500-MS Ion Trap (IT) mass spectrometer (MS), equipped with electrospray ion source (ESI) operating in negative mode [ESI(-)]. A TSKgel Amide-80 column (3.5 μm, 2.1 × 150 mm; Tosoh Bioscience, Tokyo, Japan) was used as stationary phase. A T-connector was fitted out of the chromatographic column and the effluent was split to the FLD and the MS. 0.5% formic acid in acetonitrile (A) and 1% formic acid in water (B) were used as mobile phase. Gradient elution was as follows: 0 min, 90% A; 20 min, 80% A; 25 min, 80% A; 45 min, 35% A; 51 min, 35% A. Column was left to re-equilibrate to initial conditions for 9 min. Flow rate was 200 μL/min and injected volume was 20 μL. Regarding FLD parameters, excitation wavelength was set at 231 nm and fluorescence emission at 320 nm. The MS parameters were as follows: spray chamber temperature, 50 °C; nebulizer gas pressure, 25 psi; drying gas pressure, 25 psi; drying gas temperature, 385 °C; needle voltage, ± 4500 V; spray shield voltage, 600 V. Mass spectra were acquired in negative mode in the spectral range 150–2000 Da. Being the standards of oligomeric PACs not commercially available, PAC-B2 dimer was used as standard reference compound for PACs quantification. The calibration curve was built in the range 8.5–85 μg/mL with a linear regression, as follows: *y* = 14.756*x* + 9.8284 (R^2^ = 0.9998). LOD = 0.5 µg/mL, LOQ = 5 µg/mL.

### 2.7. HPLC-DAD-MS^n^ Analysis of Secondary Metabolites

Screening of secondary metabolites from dried larch bark and LBE was performed by HPLC-DAD-MS^n^. Tentative identification of phytoconstituents was achieved by comparing their fragmentation patterns with literature data and with standard compounds, when available. The dried extract was dissolved in MeOH at a concentration of 1 mg/mL, and the solution was filtered through a 0.45 µm Millipore filter. Chromatographic separation was achieved using an Agilent 1260 binary pump equipped with autosampler and connected to a Varian 500 IT MS. A Synergi Polar RP (3 × 150 mm, 4 µm) column (Phenomenex, Torrance, CA, USA) was used as stationary phase, and a gradient mixture of methanol (A) and 1% formic acid in water (B) as mobile phase. The gradient was as follows: 0 min, 5% A; 1.5 min, 5% A; 15 min, 85% A, 20 min 100% A and isocratic up to 26 min. Re-equilibration time was 5 min. Flow rate was 0.4 mL/min. MS data were acquired in [ESI(-)]. MS parameters were as follows: needle voltage, 4500 V; capillary voltage, 70 V; RF loading, 100%; nebulizing gas pressure, 20 psi (nitrogen); drying gas pressure, 15 psi; drying gas temperature, 350 °C. *m*/*z* range was 50–2000. The turbo detection data scanning (TDDS^®^) function of the instrument was used to monitor the fragmentation patterns of eluted compounds, setting *n* = 3 levels of fragmentation. For semi-quantification of flavonoids and phenolic acids, calibration curves of rutin, gallic acid and chlorogenic acid, respectively, were obtained using concentrations ranging from 10 to 100 µg/mL. Absorption wavelengths for the detection and quantification of gallic acid derivatives, phenolic acids and flavonoids were adjusted to 280 nm, 325 nm and 350 nm, corresponding to the absorption *maxima* of gallic acid, chlorogenic acid and rutin, respectively. The calibration curves were as follows: rutin, *y* = 16.959*x* + 7.7846 (R^2^ = 0.999); chlorogenic acid, *y* = 26.97*x* + 1.4129 (R^2^ = 0.999); gallic acid, *y* = 31.47*x* + 110.01 (R^2^ = 0.997. Quantitative results were expressed as % *w*/*w*.

### 2.8. Statistical Analysis

All experiments were performed in triplicate and the data are presented as mean ± standard deviation (SD) values. GraphPad Prism^®^ software (version 6.0; GraphPad Inc., San Diego, CA, USA) was used for statistical analyses. The analysis of variance (ANOVA) and Tukey’s multiple comparisons test were used to determine statistically different values at a significance level of *p* < 0.05.

## 3. Results

### 3.1. Assessment of LBE Cytotoxicity and Acute Toxicity In Vivo

**Figure 1:**
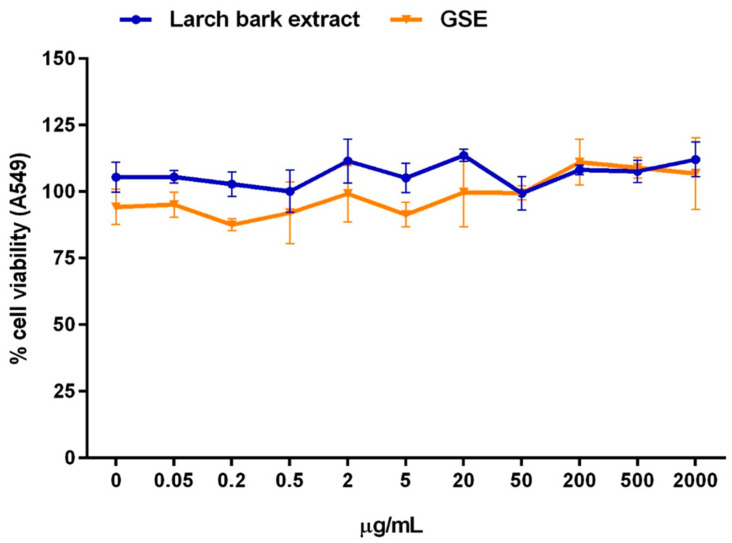
Effect of a 24 h treatment with larch bark extract (blue line) in the concentration range 0–2000 µg/mL on human A549 cells (adenocarcinomic alveolar basal epithelial cells) viability. A comparison with grapefruit seed extract (GSE; orange line) is reported. Results are the mean ± SD of *n* = 3 experiments and are expressed as percentage of absorbance of treated cells related to control.

Results from cytotoxicity assay showed that LBE in concentration range 0.05–2000 µg/mL was not reducing the viability of alveolar basal epithelial cells (Figure 1).

Regarding the in vivo acute toxicity assessment, all animals survived until the end of the study without showing any LBE-related signs of toxicity. Throughout the 15-day observation period, the weight gain of the animals was within the normal range of variation for this strain (data are reported in Supplementary Table S1). At necropsy, no macroscopic findings were observed in any animal of any step. Overall, under the conditions of the present study, a single oral application of LBE to rats at a dose of 2000 mg/kg body weight was not associated with signs of toxicity or mortality. The median lethal dose (LD_50_) of LBE after a single oral administration to female rats, observed over a period of 15 days, was >5000 mg/kg body weight.

### 3.2. Antimicrobial Activity of LBE

**Figure 2:**
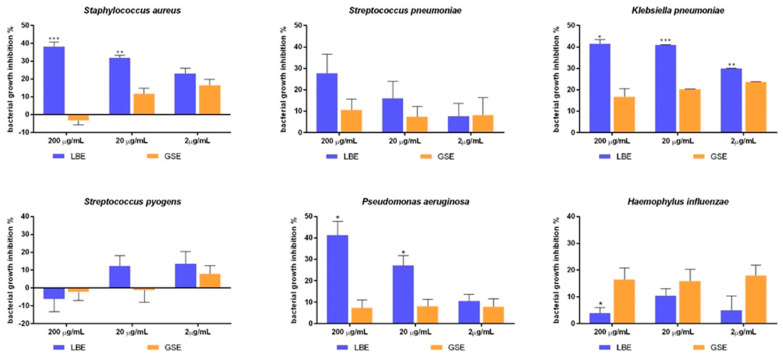
Bacterial growth inhibition % of respiratory tract pathogens treated with larch bark extract (LBE; blue bars) and grapefruit seed extract (GSE; orange bars) at different concentrations (200-20-2 µg/mL). Results are the mean ± SD of *n* = 3 experiments. * *p* < 0.05; ** *p* < 0.01; *** *p* < 0.001 vs. bacteria treated with GSE.

Results from the evaluation of LBE antimicrobial activity against respiratory-tract pathogens are reported in Figure 2. Regarding the activity on MRSA, LBE inhibited bacterial growth by 38.13% and 31.87% at the concentration of 200 and 20 µg/mL, respectively, whereas by 21.1% at 2 µg/mL. At 2 and 20 µg/mL, GSE showed a lower anti-MRSA activity compared to LBE, while at the highest dose GSE slightly increased bacterial growth.

Focusing on *S. pneumoniae*, LBE significantly inhibited pathogen growth at 200 µg/mL (22% inhibition), with higher efficacy than GSE (10.6% inhibition). LBE at 20 µg/mL retained a discrete growth inhibition activity (16%), while at 2 µg/mL the inhibitory activity was barely detectable (7% inhibition). However, GSE lacked significant inhibitory activity already at 20 µg/mL. Considering *K. pneumoniae*, LBE showed a significant growth inhibitory capacity at all concentration tested, reducing pathogen growth by 41.2%, 40.9% and 30%, respectively. GSE reduced bacterial growth to a significantly lower degree (by 17–23%).

LBE significantly inhibited *P. aeruginosa* growth at 200 µg/mL (by 42%) and 20 µg/mL (28%), while at 2 µg/mL a modest activity was detected (by 10%). On the contrary, GSE showed no significant anti-microbial activity against *P. aeruginosa* at all concentrations tested.

Considering *H. influenzae*, LBE did not significantly influence microbial growth at any concentration tested, while GSE exhibited a discrete inhibitory activity (15%) at all the dosages tested. For neither extracts a dose-response correlation was observed.

Neither LBE nor GSE demonstrated a significant antimicrobial activity on *S. pyogenes* at any concentration tested.

### 3.3. HPLC-FLD-MS Analysis of Larch Bark Procyanidins

**Figure 3:**
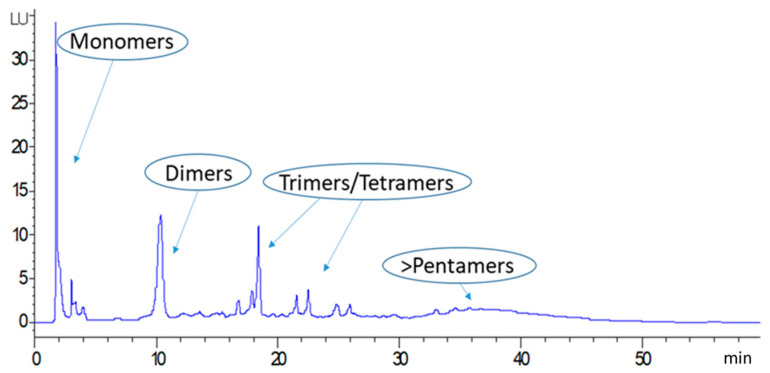
HPLC-FLD chromatogram of larch bark procyanidins. Peaks corresponding to monomers, dimers and higher-grade oligomers are indicated in the Figure.

The total amount of PAC-A and PAC-B in LBE and their degree of polymerization were assessed by HPLC-FLD-MS in both LBE and in dry larch bark powder. On the basis of the MS data, the chromatogram was divided in four parts, corresponding to monomers (PACs flavan-3-ols units consisting in catechins or epicatechins) and to the different PACs polymeric classes (dimers-hexamers) (Figure 3). 

The total amount of PACs in LBE presented a heterogeneous polymeric class composition and was around 16%, six times higher compared to dry larch bark powder (Table 1).

**Figure 4:**
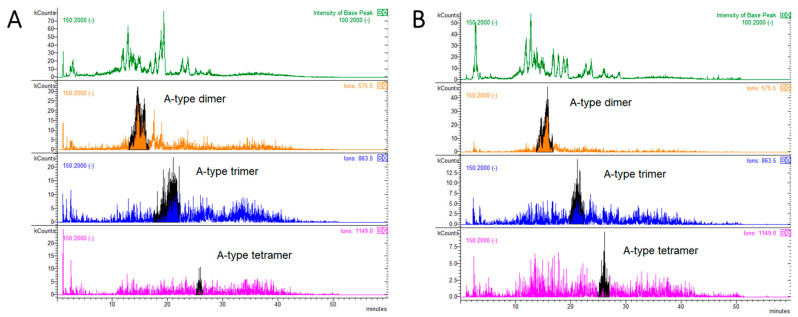
HPLC-MS chromatograms showing the distribution of type-A procyanidins in dry larch bark powder (panel **A**) and larch bark extract (panel **B**).

**Figure 5:**
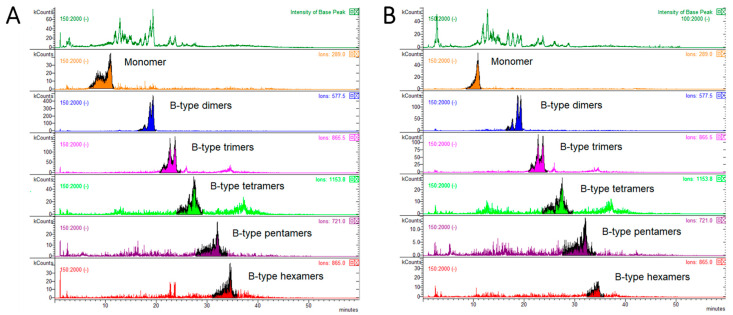
HPLC-MS chromatograms showing the distribution of type -B procyanidins in dry larch bark powder (panel **A**) and larch bark extract (panel **B**).

MS analysis was performed to distinguish between type-A and type-B PACs, and specific *m*/*z* values were monitored. Epicatechin was monitored at *m*/*z* 289. For dimers-tetramers with one or more ether bonds corresponding to type-A PACs [29], [M-H]^−^ ions at *m*/*z* 575 were monitored for dimers, *m*/*z* 863 for trimers, *m*/*z* 1149 and *m*/*z* 1151 for tetramers, and *m*/*z* 1437 and *m*/*z* 1727 for pentamers and hexamers, respectively. For those PACs with no ether bonds, corresponding to B-type PACs, [M-H]^−^ ions at *m*/*z* 577, *m*/*z* 865 and *m*/*z* 1153 were monitored for dimers, trimers and tetramers, respectively, while [M-2H]^2−^ ions at *m*/*z* 721 and 865 were monitored for pentamers and hexamers, respectively. The relative percentages of PAC-A and PAC-B oligomers and PAC-A/PAC-B ratios of dried larch bark powder and LBE are reported in Table 2, while the respective HPLC-MS chromatograms are reported in Figure 4 and Figure 5.

The most evident difference between the larch bark powder and the extract in matter of PAC-A and PAC-B composition resided in the ratio between the two types of PAC (PAC-A:PAC-B oligomers = 0.17 in larch bark powder; 0.10 in larch bark extract). The higher amount of PAC-A in the dried powder is due mainly to the higher percentage of dimers compared to the extract (Table 2). On the other hand, the relative % of PAC-B dimers appeared to be higher in LBE, while PAC-B trimers were present at lower %. Higher MW oligomers appeared to be comparable between the larch bark powder and the extract (Table 2).

### 3.4. HPLC-DAD-MS^n^ Analysis of Secondary Metabolites

The amounts of polyphenols in both larch bark powder and LBE were determined by HPLC-DAD and are reported in Table 3. The results show that the total amount of phenolic compounds in LBE were almost seven times higher than dry larch bark powder, as expected for a dry hydro-alcoholic extract, in which phenolic constituents are highly concentrated if compared to the plant material. Qualitative results from HPLC-MS^n^ phytochemical screening of both larch bark powder and LBE are reported in Table 4. Several tentatively identified metabolites are here reported in larch bark for the first time. Overall, the phytochemical profiles of larch bark powder and LBE were not completely matching, being some metabolites detected in either one or the other matrix. This is due to the higher concentration of metabolites in LBE, that allows to detect a higher fraction of compounds from the HPLC-MS data. On the other hand, in view of the complexity of the plant material, extraction of the bark powder using a single solvent (MeOH in this case) leads to a selective extraction of certain compounds, leading to a different composition of the extract respect to the raw matrix. In general, a higher percentage of flavonoids and phenolic acids was identified in the bark powder, together with catechin and its isomer epicatechin, a stilbene derivative and fatty acids (Table 4). Characteristic phenols of larch bark were also detected, namely the spiropolyphenols larixinol and epilarixinol [3]. On the other hand, apart from larixinol and epilarixinol, other two spiropolyphenols characteristic of *Larix* species [3] were detected in LBE. Compared to the dry larch bark powder, fewer flavonoids and phenolic acids were identified in LBE, although the content of total flavonoids, phenolic acids and gallic acid derivatives was higher (Table 3).

## 4. Discussion

In our previous work on larch bark extracts, different ethanol/water mixtures (0°, 40°, 60°, 80° alcoholic grade) were assessed in laboratory scale to select the most efficient solvent for flavonoids and PACs extraction [3]. Water and ethanol were used as solvents because they are considered natural, environmentally friendly (green solvents), nontoxic and food-grade [34], and these are important aspects to consider while developing natural extracts with potential pharmaceutical and nutraceutical applications. In all the extracts obtained, PAC-B were reported as major compounds, while other phenolic constituents such as flavonoids and catechins were more abundant in extracts obtained with 40° and 60° ethanol [3]. Here, the laboratory-scale maceration protocol was transferred to a pilot plant-scale for a larger industrial application. Based on our previous results [3] and in order to preserve extraction efficiency reducing as much as possible the environmental impact, 40° ethanol was maintained as extraction solvent, and the extraction yield was 10%.

The obtained extract was tested in vitro at different dosages (2–200 µg/mL) for its antibacterial effects against different Gram-negative and Gram-positive species responsible for common respiratory-tract infections, and results were compared with those obtained testing a commercial GSE against the same strains. The tested LBE dosages fell within the dosage range that could be considered safe for oral ingestion, as demonstrated by the cytotoxicity assay in vitro on alveolar epithelial cells and in rats monitored for 15 days after a single LBE ingestion (2000 mg/kg body weight). Data from antibacterial assays showed that LBE was more effective in inhibiting the growth of MRSA, *S. pneumoniae*, *K. pneumoniae*, and *P. aeruginosa* than GSE, suggesting its potential application as active ingredient for the prevention of respiratory-tract infections. GSE was chosen as comparison due to its documented antibacterial activity against several Gram-negative and Gram-positive species [35,36,37], and because this natural extract is currently used in food supplements and nutraceuticals despite there are doubts about the chemical constituents responsible of these effects. In fact, although a recent publication indicates that the flavonoid naringin together with other unidentified phenolic compounds could be responsible, at last in part, of the antimicrobial activity of GSE [37], older works reported the detection of quaternary ammonium salts preservatives such as benzethonium [38,39,40] and benzalkonium chlorides [41] in commercial GSE, which have been claimed to be formed by chemical conversion of GSE polyphenols during its production [35]. However, there is a general scientific disagreement about the supposed chemical pathways leading to the production of such ammonium quaternary salts from natural constituents of grapefruit seeds, considering also that the methods of GSE production are proprietary and have not been completely specified [42]. Furthermore, there is no clear evidence that these compounds are responsible of the antibacterial activity of GSE. Nevertheless, despite all these issues and the lack of exhaustive characterizations of GSE bioactives [26], this natural extract is commercially available and currently used in formulations for human use.

In this work, we propose LBE as a possible alternative to GSE for pharmaceutical and nutraceutical applications. After extraction of larch bark, we performed the characterization of PACs profile and total phenolic content in the dry extract by using HPLC-FLD-MS. The extraction procedure allowed to concentrate almost six times the total PACs content in the dried bark, reaching an amount of almost 16% *w*/*w* in the final extract. Monomers and higher MW oligomers (i.e., pentamers and hexamers) contributed the most to this amount, representing the 5.12% and 5.28% *w*/*w* of the final extract, respectively. Regarding the composition in type-A and type-B PACs, the extraction procedure allowed to reach a PAC-A/PAC-B ration in LBE of 0.10, compared to 0.17 in the dried larch bark. In LBE, type-B PACs represented the 90.72% of total PACs, being dimers and trimers the most representative oligomers (Relative abundance = 49% and 23.19%, respectively). The abundance of PACs in LBE and the peculiar PAC-A and PAC-B oligomers distribution could in part explain the observed antibacterial activity against MRSA, *S. pneumoniae*, *K. pneumoniae*, and *P. aeruginosa*. PACs from larch (*L. gmelinii*) have shown to effectively inhibit the growth of *S. aureus* at a MIC of 1.75 mg/mL [18]. In the same study, the authors showed that larch PACs could exert their effect on *S. aureus* by altering several morphological properties of the bacterial membrane, as well as increasing the membrane electrolytic transportation, binding to DNA grooves to form complexes, and affecting energy metabolic systems and protein expression [18]. B-type PACs have also been associated to the antimicrobial activity of different bark, wood, stems and leaves extracts from *Uncaria tomentosa* against both *S. aureus* and *P. aeruginosa* [43], while the high PACs content of PACs-enriched grape-seed extracts has been associated to their bacteriostatic activity against *P. aeruginosa*, *Streptococcus* spp. [44], *S. pyogenes*, and *H. influenzae* [45].

Results from HPLC-DAD-MS characterization of LBE phenolic profile revealed a total phenolic content of 3.84% *w*/*w*, almost seven times higher than dry larch bark powder. Major contributors were gallic acid derivatives (1.57%) and phenolic acids, accounting for 1.66% *w*/*w* of LBE. Together with the high PACs content, the phenolic composition of LBE could explain, at least in part, the antibacterial activity observed in vitro. In fact, gallic acid derivatives, phenolic acids and flavonoids have all been reported to exert effective antimicrobial activity against different bacterial species [46,47,48], among which several affecting the upper respiratory tract [49]. More specifically, the flavonoid isoquercitrin has been recently reported to significantly reduce the activity of staphylocoagulase, a virulence factor of *S. aureus* considered a promising target for the treatment of *S. aureus* infections, and to reduce the bacterial burden, pathological damage, and inflammation of lung tissue in mice [50]. Another flavonoid identified in LBE, rutin, has been already reported as antimicrobial agent, and in vitro antibacterial activity against *S. aureus* and *P. aurigenosa* has been documented [51]. Also stilbene derivatives have shown to exert antimicrobial activity against respiratory tract pathogens, as in the case of piceatannol, for which the activity against *S. aureus* (MIC = 25 µg/mL) has been reported [52]. Overall, although these preliminary data can partially associate the antibacterial activity of LBE observed in vitro to its chemical composition, further studies are required to correlate specific constituents to the efficacy against different bacteria.

## 5. Conclusions

In this work, we reported the antimicrobial activity of a standardized larch bark dry hydro-alcoholic extract against several respiratory-tract pathogens, among whom the commonly diffused *S. aureus*, *K. pneumoniae* and *P. aeruginosa*. Larch bark is a by-product accumulated during timber production in the sawmilling industry, and, in spite of its content in potentially bioactive compounds, it has been scarcely evaluated as potential bioactive ingredient. In this study, LBE was produced in an industrial pilot plant using an environmentally-friendly extraction method, suitable for the production of pharmaceutical and nutraceutical grade extracts. The ethanol/water mixture and the extraction protocol employed allowed us to obtain a LBE with an amount of PACs almost six times higher than obtained from dried bark powder, reaching 15% *w*/*w*. The high amount of PACs could be considered as the main contributor to the observed antibacterial activity of LBE, together with other phenolic compounds that were identified, namely the flavonoids isoquercitrin and rutin, and the stilbene piceatannol 3′-O-glucoside. In conclusion, the LBE obtained with an environmental-friendly extraction procedure represents a sustainable source of valuable bioactive compounds, and antimicrobial assays confirmed that this bio-waste could be a promising antibacterial ingredient for pharmaceutical and nutraceutical applications.

## Figures and Tables

**Table 1:** Amounts of different PACs polymeric classes in larch bark dry powder and larch bark extract. Data were obtained by HPLC-FLD analysis and are expressed as %*w*/*w* ± SD.

Larch Sample	PACs % *w*/*w*				
	Mon	Dim	Tri/Tetr	Pent/Hex	Total
Dry larch bark powder	0.93 ± 0.07	0.36 ± 0.03	0.38 ± 0.06	0.91 ± 0.01	2.58
Larch bark extract	5.12 ± 0.10	3.21 ± 0.06	2.32 ± 0.18	5.28 ± 0.24	15.92

**Table 2:** Relative PAC-A and PAC-B % and PAC-A/PAC-B ratio related to polymerization degree of PACs contained in dry larch bark powder and in larch bark extract. Data were obtained by HPLC-MS analysis and are expressed as % *w*/*w* ± SD.

Sample	Polymerization Degree	Relative%			PAC-A/PAC-B Ratio
		PAC-A	PAC-B	PAC-A + PAC-B	
**Dry larch bark powder**	Dimers	10.29 ± 0.23	33.55 ± 0.63	43.83 ± 0.86	0.31 ± 0.00
	Trimers	3.38 ± 0.09	32.69 ± 0.11	36.07 ± 0.21	0.10 ± 0.00
	Tetramers	0.88 ± 0.05	9.14 ± 0.06	10.02 ± 0.01	0.09 ± 0.01
	Pentamers	-	4.81 ± 0.08	4.81 ± 0.08	-
	Hexamers	-	4.68 ± 0.08	4.68 ± 0.08	-
	Total %	14.60	85.40	100.00	0.17
**Larch bark extract**	Dimers	5.03 ± 0.10	49.00 ± 0.02	54.02 ± 0.08	0.10 ± 0.00
	Trimers	3.72 ± 0.10	23.19 ± 0.10	26.92 ± 0.21	0.16 ± 0.00
	Tetramers	0.52 ± 0.01	8.89 ± 0.04	9.41 ± 0.03	0.06 ± 0.00
	Pentamers	-	4.79 ± 0.02	4.79 ±0.02	-
	Hexamers	-	4.76 ± 0.02	4.76 ± 0.02	-
	Total %	9.28	90.72	100.00	0.10

**Table 3:** Quantitative characterization of phenolic profile in dry larch bark powder and larch bark extract.

Sample	% *w*/*w* (±SD)			
	Gallic Acid Derivatives (GAE)	Phenolic Acids (CAE)	Flavonoids (RE)	Total PolyPhenols
Dry larch bark powder	0.22 ± 0.01	0.29 ± 0.01	0.05 ± 0.01	0.56
Larch bark extract	1.57 ± 0.04	1.66 ± 0.06	0.61 ± 0.02	3.84

GAE: Gallic acid equivalent; CAE: chlorogenic acid equivalent; RE: rutin equivalent.

**Table 4:** Secondary metabolites tentatively identified in dry larch bark powder and in larch bark extract by HPLC-MS^n^.

Molecular Ion (*m*/*z*)	MS2 Main Fragments *	MS3 Main Fragments	R.T. (min)	Tentative Identification	Larch Bark Powder **	Larch Bark Extract **	Chemical Class	Refs.
301	179 151		11.7	Quercetin	D	ND	Flavonoid	[30]
447	285		10.7	Kaempferol 3-β-D-glucopyranoside	D	D	Flavonoid	[31]
449	287		11.8	Unknown flavonoid glucoside	D	ND	Flavonoid	[32]
463	301	179 151	10.3	Isoquercitrin	D	D	Flavonoid	[32]
505	301	179 151	10.4	Quercetin 3-(2″-acetylgalactoside)	D	D	Flavonoid	[33]
609	301		10.0	Rutin ^#^	D	D	Flavonoid	-
511	483 385 267		11.2	4,4′,6′-trihydroxy-2,2′-bis(4-hydroxyphenyl)-2H,2′H-spiro(benzo(1,2-b:3,4-b′)difuran-8,3′-benzofuran)-7(3H)-one	ND	D	Spiro-polyphenol	[3]
541	513 497 415	309 281	10.4	Larixinol	D	D	Spiro-polyphenol	[3]
541	513 497 415	309 281	10.8	Epilarixinol	D	D	Spiro-polyphenol	[3]
673	511	483 385 267	10.7	2′-Caffeoyl-4,4′,6′-trihydroxy-2-bis(4-hydroxyphenyl)-2H,2′H-spiro[benzo[1,2-b:3,4-b′]difuran-8,3′-benzofuran]-7(3H)-one	ND	D	Spiro-polyphenol	[3]
353	191 179 173		4.4	Caffeoylqunic acid isomer	D	ND	Phenolic acid	[30]
353	191 179 173		6.6	Chlorogenic acid ^#^	D	ND	Phenolic acid	[30]
353	191 179 173		7.9	Caffeoylqunic acid isomer	D	ND	Phenolic acid	[30]
863	739 713 695 577		7.7	Procyanidin trimer B	D	D	Procyanidin	[3]
863	739 713 695 577		8.1	Procyanidin trimer B	D	D	Procyanidin	[3]
863	739 713 695 577		8.6	Procyanidin trimer B	D	D	Procyanidin	[3]
289	245 205 179		6.9	Catechin ^#^	D	D	Catechin	-
289	245 205 179		8.1	Epicatechin ^#^	D	D	Catechin	-
405	243	225 201 175	9.7	trans-Piceatannol 3′-O-glucoside	D	D	Stilbene	[31]
405	243	225 201 175	10.4	cis-Piceatannol 3′-O-glucoside	ND	D	Stilbene	[31]
191	111		2.4	Quinic acid	D	D	Others	[3]
327	291 229 211 183 171		12.3	Oxo-dihydroxyoctadecenoic acid	D	ND	Others	[30]

* Values in bold indicate the base peak. ** D: detected compound; ND: not detected compound. ^#^ Identified by comparison with reference standard.

## Data Availability

Not applicable.

## Supplementary Materials

The following are available online at https://www.mdpi.com/article/10.3390/antibiotics10070789/s1: Table S1. Body weights (BW; g) of female Wistar rats (*n* = 6) employed to perform the acute toxicity test of LBE (dose = 2000 mg/kg body weight). Data were acquired on day 1 (prior to LBE administration), 8 and 15 (end of experiment).
