# Supplementary material for: Polyphenol-Rich Larix decidua Bark Extract with Antimicrobial Activity against Respiratory-Tract Pathogens: A Novel Bioactive Ingredient with Potential Pharmaceutical and Nutraceutical Applications
Source: Antibiotics (Basel). 2021 Jun 28;10(7):789. doi: 10.3390/antibiotics10070789 (PMC8300756; doi:10.3390/antibiotics10070789)
Supplement: Supplementary file 1 [file antibiotics-10-00789-s001.zip › antibiotics-1257115-supplementary.pdf]

Supplementary Methods

HPLC-DAD Method for Analysis of Grapefruit Seed Extract (GSE)

Two hundred milligrams of GSE were suspended in 25 mL of methanol:water (50:50) and were extracted using an ultrasound bath for 20 min. Samples were centrifuged at 13,000 rpm for 10 min and liquids were collected in vial for analysis.

The amount of glycoside flavanones was quantified by HPLC-DAD. The chromatographic system was composed of an Agilent 1260 series quaternary pump coupled to an Agilent 1260 diode array detector (DAD). An Agilent Eclipse C8 column (5 µm, 4.6 × 150 mm) was used as stationary phase. Acetonitrile (A) and 0.1% formic acid in water (B) were used as mobile phase. Gradient elution was as follows: 0 min, 10% A; 30 min, 100% A; 35 min, 100% A; 36 min, 10% A; 40 min, 10% A. Flow rate was 1 mL/min and injected volume was 10 µL. Regarding DAD parameters, wavelength was set at 280 nm. Hesperidin 98% (Sigma-Aldrich) was used as standard reference compound for glycosidic flavanones quantification in GSE sample. Calibration curve was built in the range 10-100 µg/mL with a linear regression, as follows: $y = 15.415x – 9.9204$; $R^2 = 0.999$; LOD = 1.5 µg/mL, LOQ = 5 µg/mL.

Table S1. Body weights (BW; g) of female Wistar rats (n = 6) employed to perform the acute toxicity test of LBE (dose = 2000 mg/kg body weight). Data were acquired on day 1 (prior to LBE administration), 8 and 15 (end of experiment).

Step	Animal No. / Sex	Starting Dose (mg/kg bw)	BW (g)			Body Weight Change in Comparison to Day 1 (%)
			Day 1	Day 8	Day 15	Day 15
1	1 / Female	2000	174	205	216	24
	2 / Female		169	203	214	27
	3 / Female		153	180	190	24
2	4 / Female		185	204	205	11
	5 / Female		169	196	200	18
	6 / Female		167	201	215	29
